# Respiratory muscle endurance training among patients with chronic diseases: A systematic review of available evidence

**DOI:** 10.1113/EP092300

**Published:** 2025-01-06

**Authors:** Hélène Laurent, Frédéric Costes, Ruddy Richard, Marc Filaire

**Affiliations:** ^1^ Centre de Lutte Contre le Cancer Clermont Auvergne Métropole Centre Jean Perrin Service de Soins Oncologiques de Support Clermont‐Ferrand France; ^2^ Université Clermont Auvergne Clermont‐Ferrand France; ^3^ CHU Clermont‐Ferrand, Service de Médecine du Sport et des Explorations Fonctionnelles Clermont‐Ferrand France; ^4^ Centre de Recherche en Nutrition Humaine Clermont‐Ferrand France; ^5^ Centre de Lutte Contre le Cancer Clermont Auvergne Métropole Centre Jean Perrin Service de Chirurgie Thoracique et Endocrinienne Clermont‐Ferrand France

**Keywords:** effectiveness, isocapnic hyperventilation, respiratory endurance, review, voluntary isocapnic hyperpnoea

## Abstract

This systematic review summarizes the available evidence on respiratory muscle endurance training involving voluntary isocapnic hyperpnoea among patients with chronic diseases. It includes both randomized and non‐randomized controlled trials implementing this exercise training modality performed either alone or in combination with other interventions. It was conducted using the following databases: PubMed, Google Scholar, Physiotherapy Evidence Database (PEDro), Embase, CINAHL, CENTRAL, Cochrane and ReeDOC. It was drafted in accordance with the PRISMA guidelines. The final analysis was conducted on 12 studies (*n* = 257). There was heterogeneity in participants, training modalities and comparators used. The underpowered level of evidence is attributable to the lack of robustness of the original studies, including a lack of description of the intervention, lack of blinding, and missing data. Respiratory muscle endurance training is an exercise training modality that is both safe and feasible, even in the setting of the patient's home. It increases respiratory endurance time. However, its effect on peak oxygen consumption at exercise, maximal work rate, 6‐min walking distance, quality of life, dyspnoea and fatigue remains to be confirmed. In conclusion, this systematic review shows that respiratory muscle endurance training increases respiratory endurance among patients with chronic diseases. The populations that benefit the most and the mechanisms involved remain to be investigated. Further high‐quality studies are needed to understand its role, whether it is performed alone or as an add‐on modality to usual pulmonary rehabilitation programmes.

## Abstract (highlights)

- **What is the topic of this review?**
  The aim was to summarize evidence on respiratory muscle endurance training involving voluntary isocapnic hyperpnoea among patients with chronic diseases.
- **What advances does it highlight?**
  This systematic review summarizes the available evidence on respiratory muscle endurance training among patients with chronic diseases. It shows that this exercise training modality increases respiratory endurance. The populations that benefit the most and the mechanisms involved remain to be investigated. Further high‐quality studies are needed to understand its role, whether it is performed alone or as an add‐on modality to usual pulmonary rehabilitation programmes.

## 1 INTRODUCTION

The measurement of maximal respiratory pressures and maximal voluntary ventilation (MVV) are common laboratory procedures, used to assess chest wall mechanical properties and function (American Thoracic Society/European Respiratory Society, 2002; Laveneziana et al., 2019). These tests are useful in detecting reductions in respiratory strength and/or endurance and in determining the appropriate intensity for respiratory muscle training.

In patients with chronic obstructive pulmonary disease (COPD), the respiratory muscles are subjected to an increased workload, owing to hyperinflation and increased intrinsic positive end‐expiratory pressure (Decramer et al., 1980; Rochester et al., 1979). This can result in a reduction in respiratory strength and/or endurance, as well as in addition to respiratory muscle fatigue (Decramer et al., 1980; Rochester et al., 1979). Patients with COPD experience remodelling of the respiratory muscles as a result of molecular and/or structural adaptations. Biopsies have revealed that there is a greater degree of atrophy in type II fibres, which is accompanied by disruption of the sarcomere (Hughes et al., 1983; Orozco‐Levi et al., 2001). This results in a reduction in the force‐generating capacity of individual diaphragm muscle fibres, which is associated with increased proteolysis (Ottenheijm et al., 2005). Consequently, patients can demonstrate a reduced exercise tolerance, particularly those who experience dyspnoea or dynamic pulmonary hyperinflation during exercise (Aaron et al., 1992; Dempsey et al., 2006; O'Donnell et al., 2020; Romer & Polkey, 2008; Sheel, 2002).

The evidence indicates that targeted respiratory muscle training can be an effective method for managing increased workload and improving respiratory function. It has been demonstrated that respiratory muscle training can result in structural adaptations of the respiratory muscles. Indeed, an increase in diaphragm thickness has been observed (Chiappa et al., 2008). There has been ∼38% increase in the proportion of type I fibres and ∼21% increase in the size of type II fibres in the external intercostal muscles (Ramirez‐Sarmiento et al., 2002). Appropriate respiratory muscle training programmes could result in an increase in respiratory muscle strength and/or endurance (Leith & Bradley, 1976; Shei et al., 2021). Ultimately, these improvements could lead to a reduction in the perception of breathlessness and respiratory exertion during exercise, as well as in addition to an improvement in exercise performance following respiratory muscle training (Verges et al., 2008).

From a practical point of view, inspiratory threshold loading, flow resistance loading and voluntary isocapnic hyperpnoea are the main modalities used for respiratory muscle training. The first two methods are strength oriented and consist of breathing against a pressure threshold resistance or a flow resistance loading twice a day, for 5–7 days a week, during 4–12 weeks, at 30%–40% of maximal inspiratory pressure (MIP) (Beaumont et al., 2018; Caicedo‐Trujillo et al., 2023; Ge et al., 2018; Tórtola‐Navarro et al., 2022). The evidence clearly demonstrates that inspiratory muscle training (IMT) has a positive impact on a number of key outcomes for patients with COPD. These include improvements in MIP, 6‐min walking distance (6MWD), quality of life (QoL), and dyspnoea (Beaumont et al., 2018; Gosselink et al., 2011; Lötters et al., 2002). Furthermore, the addition of IMT to pulmonary rehabilitation (PR) has been shown to have an additional effect on MIP, while having no effect on 6MWD or dyspnoea (Beaumont et al., 2018; Gosselink et al., 2011; Lötters et al., 2002). Patients with COPD and inspiratory muscle weakness (MIP of <60 cmH_2_O) are more likely to increase both MIP and 6MWD when IMT is added to PR (Gosselink et al., 2011; Lötters et al., 2002). In cardiothoracic and abdominal surgery, IMT has been shown to increase MIP, while decreasing the incidence of pulmonary postoperative complications (PPCs) and the length of hospital stay (Ge et al., 2018; Hulzebos et al., 2006; Nomori et al., 1994; Weiner et al., 1997). The third method, known as respiratory muscle endurance training (RMET), is specifically endurance oriented. It consists of daily sessions of voluntary isocapnic hyperpnoea, performed 5–7 days a week, during 3–14 weeks, at 50%–60% of MVV (Koppers et al., 2006; Laurent et al., 2020; Villiot‐Danger et al., 2011). Furthermore, a positive and significant correlation has been identified between maximal respiratory pressures and respiratory endurance time (RET) (Vincent et al., 2016). However, it is not possible to predict respiratory muscle endurance accurately in relationship to fatigue resistance based on maximal respiratory muscle strength, nor can it be trained specifically with IMT. Moreover, hyperventilation provides a more physiological exercise modality for training the respiratory muscles. Finally, the efficacy of RMET programmes in increasing respiratory endurance has been demonstrated in studies recruiting patients with chronic diseases (Budweiser et al., 2006; Koppers et al., 2006; Laurent et al., 2020; Rassler et al., 2007; Villiot‐Danger et al., 2011). Nonetheless, there is currently no convincing evidence to suggest that RMET is an effective modality for improving exercise capacity, QoL, dyspnoea or fatigue. Furthermore, no systematic review has been conducted to date. A systematic review was therefore conducted with the specific aim of summarizing the available evidence on RMET performed with voluntary isocapnic hyperpnoea among patients with chronic diseases.

## 2 MATERIALS AND METHODS

This systematic review was registered on the Prospero website (CRD42022334822). It was drafted in accordance with the PRISMA guidelines (Page et al., 2021).

### 2.1 Inclusion and exclusion criteria

This systematic review included both randomized controlled trials (RCTs) and non‐RCTs. Abstracts, retrospective studies, editorials, letters and case reports were excluded from the review.

The studies were selected based on their alignment with the population, intervention, comparator and outcome (PICO) criteria. To be included in the analysis, articles had to recruit patients with a chronic disease, defined as a disease or condition that usually lasts for a minimum of 3 months and may deteriorate over time. Studies that recruited healthy or trained subjects were excluded from the review. The intervention under consideration was RMET performed either alone or in combination with other interventions. The RMET modality was defined as voluntary isocapnic hyperpnoea performed with a customized or commercially available device (Spirotiger® device, Idiag, Fehraltorf, Switzerland) or an equivalent. Studies that implemented respiratory muscle strength training, deep breathing exercises, chest physiotherapy or incentive spirometry were excluded from the review. The comparators under consideration were deep breathing exercises, incentive spirometry, IMT, endurance training/whole‐body exercise training, usual care, placebo and no intervention. The outcomes under consideration were safety (adverse events), feasibility (completion and adherence rates), RET, pulmonary function test parameters [vital capacity (VC), forced vital capacity (FVC), forced expiratory volume in the first second (FEV_1_), MIP, MEP and MVV], cardiopulmonary exercise test parameters [maximal oxygen consumption (${\dot{V}}_{O_{2}\mathrm{peak}}$) and maximal work rate (MWR)], 6MWD, exercise capacity, functional capacity, postoperative morbimortality (PPCs and length of hospital stay), QoL and symptoms (dyspnoea and fatigue).

### 2.2 Search strategy

A comprehensive search of the literature was conducted using the following databases: PubMed, Google Scholar, Physiotherapy Evidence Database (PEDro), Embase, CINAHL, CENTRAL, Cochrane and ReeDOC. The search was limited to articles published from inception to September 2024. It was limited to English and French language publications. The search equation was determined with the assistance of a librarian. It consisted of the following terms: “human”[All Fields] AND “inspiratory muscle training”[All Fields] OR “expiratory muscle training”[All Fields] OR “respiratory muscle training”[All Fields] OR “hyperpnea”[All Fields] OR “isocapnic hyperventilation”[All Fields] OR “voluntary isocapnic hyperpnea”[All Fields] AND “safety”[All Fields] OR “feasibility”[All Fields] OR “efficacy”[All Fields] OR “effectiveness”[All Fields]. To extend the search, keywords and their synonyms were combined. Their validity was determined through the use of database thesauruses and MeSH terms, with verification conducted on the CISMeF database of the Rouen University Hospital. To complete the initial search, the reference lists and keywords from the identified references were consulted to identify additional relevant sources. The lead author (H.L.) performed manual citation tracking.

### 2.3 Selection of studies and data extraction

Two readers (H.L. and M.F.) selected the references according to the established criteria, based on their titles and abstracts. They then read the full text and extracted the data in accordance with the PICO criteria. Duplicate publications were included only once. Our analysis revealed that the Clear grid (Boutron et al., 2005) and the Consort grid for non‐pharmacological trials (Boutron et al., 2008) lack sufficient discriminatory and descriptive power to collect the content of each intervention described in the literature. Instead, we developed our custom data extraction grid based on the opinions of a multidisciplinary team of clinicians with expertise in RMET, including physiotherapists, physiologists and thoracic surgeons. In the event of any discrepancies, a third reader (F.C.) was consulted to reach a consensus. The lead author (H.L.) extracted the data manually, including the characteristics of the studies (author, year of publication and design), the characteristics of the patients, the characteristics of the interventions (setting, programme duration, session duration, frequency, type and intensity), the characteristics of the comparators, and the outcome data. These data were presented in tables.

### 2.4 Methodological quality assessment

The methodological quality of the selected studies was evaluated by the lead author (H.L.). The PEDro scoring system (Verhagen et al., 1998) for RCTs and the Revised Cochrane Risk‐of‐Bias Tool for Non‐Randomized Studies of Interventions (Robins‐1) (Sterne et al., 2016) for non‐RCTs were used. Any discrepancies were resolved by a second assessor (M.F.) until a consensus was reached. The PEDro scale is an 11‐item scale with a maximal score of 10 that assesses the methodological quality of RCTs. This tool considers several key aspects of study quality, including eligibility, randomization, allocation concealment, similarity at baseline, blinding of patients, therapists and assessors, the proportion of key outcomes obtained from >85%, intention‐to‐treat analysis, between‐group statistical comparisons, and both point and variability measures. The Robins‐1 scale is an eight‐item scale that assesses the risk of bias of non‐RCTs and categorizes it as low, moderate or high. This tool considers several key aspects of study quality, including confounding, selection, classification of intervention, deviation from interventions, missing outcome data, measurement of outcome, selection of reported result, and overall risk of bias.

## 3 RESULTS

### 3.1 Search results

**FIGURE 1:**
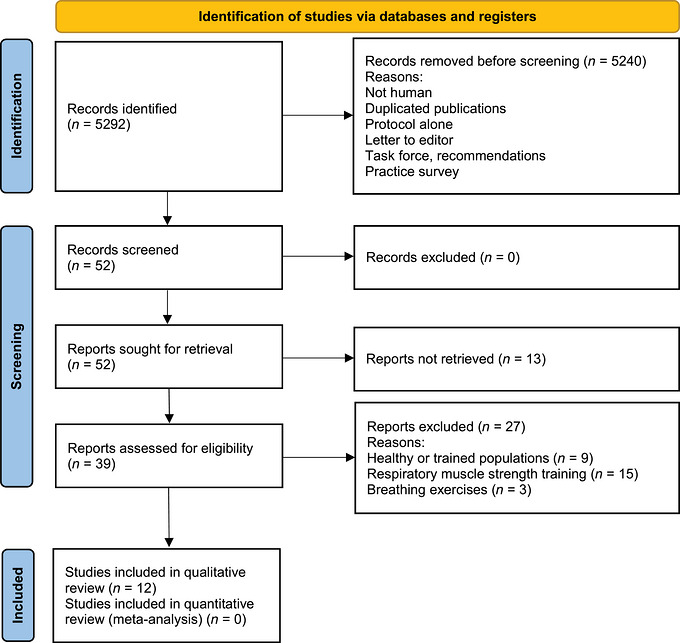
Flowchart.

The flowchart for this systematic review is presented in Figure 1. A total of 5292 reports were identified in the included databases. Of these, 52 titles and abstracts were reviewed, and 39 full‐length texts were assessed for eligibility. Twenty‐seven studies that recruited healthy or trained populations or that implemented respiratory muscle strength training or breathing exercises were excluded from analysis. The final analysis was conducted on 12 studies.

### 3.2 Characteristics and aims of included studies

The characteristics of the included studies are presented in Table 1. The review included 2 RCTs (Laurent et al., 2020; Xi et al., 2019) and 10 non‐RCTs (Budweiser et al., 2006; Freitag et al., 2018; Koppers et al., 2006; Mador et al., 2005; Rassler et al., 2007, 2011; Rigamonti et al., 2014; Salvadego et al., 2017; Scherer et al., 2000; Villiot‐Danger et al., 2011). Eight studies used explicit inclusion criteria: FEV_1_ between 30% and 80% of the predicted value or <70% of the predicted value, FEV_1_/FVC <70% of the predicted value, MIP <70% of the predicted value, inspiratory vital capacity >25% of the predicted value, body mass index standard deviation score (BMI‐SDS) above 2, BMI >30 kg/m^2^, and Oosterhuis Myasthenia Gravis classification between 1 and 3. The selected studies were designed to evaluate the safety (adverse events), feasibility (completion and adherence rates) and effect of RMET on RET, pulmonary function test parameters (VC, FVC, FEV_1_, MIP, MEP and MVV), cardio‐pulmonary exercise test parameters (${\dot{V}}_{O_{2}\mathrm{peak}}$ and MWR), exercise capacity (6MWD and constant‐load exercise endurance time), postoperative morbimortality (PPCs and length of hospital stay), QoL and symptoms (dyspnoea and fatigue).

**TABLE 1:** Study and patient characteristics from selected studies.

Author, publication year	Study design	Participants characteristics
Koppers et al. (2006)	Non‐RCT	COPD FEV_1_: 55% predicted TG: *n* = 18 CG: *n* = 18
Mador et al. (2005)	Non‐RCT	COPD FEV_1_: 44% predicted TG: *n* = 15 CG: *n* = 14
Scherer et al. (2000)	Non‐RCT	COPD FEV_1_: 51% predicted TG: *n* = 15 CG: *n* = 15
Laurent et al. (2020)	RCT	NSCLC TNM stage: I–IIIB TG: *n* = 14 CG: *n* = 12
Budweiser et al. (2006)	Non‐RCT	Restrictive thoracic disease VC: 50% predicted TG: *n* = 15 CG: *n* = 15
Rigamonti et al. (2014)	Non‐RCT	Obesity BMI: 39 kg/m^2^ *n* = 7
Salvadego et al. (2017)	Non‐RCT	Obesity BMI: 39 kg/m^2^ TG: *n* = 9 CG: *n* = 8
Villiot‐Danger et al. (2011)	Non‐RCT	Obesity BMI: 45 kg/m^2^ TG: *n* = 10 CG: *n* = 10
Freitag et al. (2018)	Non‐RCT	Myasthenia gravis TG: *n* = 18 CG: *n* = 6
Rassler et al. (2007)	Non‐RCT	Myasthenia gravis *n* = 10
Rassler et al. (2011)	Non‐RCT	Myasthenia gravis *n* = 10
Xi et al. (2019)	RCT	Spinal cord injury FVC = 58% predicted *n* = 18

Abbreviations: BMI, body mass index; CG, control group; COPD, chronic obstructive pulmonary disease; FEV_1_, forced expiratory volume in the first second; FVC, forced vital capacity; NSCLC, non‐small cell lung cancer; RCT, randomized controlled trial; TG, training group; TNM, tumour node metastasis classification; VC, vital capacity.

### 3.3 Population characteristics

The characteristics of the patients recruited in the included studies are presented in Table 1. The total sample size was 257 patients with chronic diseases. The sample sizes of included studies ranged from 7 to 36 participants, varied and were often small. The studies recruited patients with moderate to severe COPD (FEV_1_ from 44% to 55% of the predicted value; *n* = 3) (Koppers et al., 2006; Mador et al., 2005; Scherer et al., 2000), patients eligible for non‐small cell lung cancer (NSCLC) resection surgery (TNM stage from I to IIIB; *n* = 1) (Laurent et al., 2020), patients with restrictive thoracic disease treated with nocturnal non‐invasive positive‐pressure ventilation (NPPV) (VC = 50% predicted value; *n* = 1) (Budweiser et al., 2006), patients with severe obesity (BMI ≥ 35 kg/m^2^; *n* = 3) (Rigamonti et al., 2014; Salvadego et al., 2017; Villiot‐Danger et al., 2011), patients with myasthenia gravis (*n* = 3) (Freitag et al., 2018; Rassler et al., 2007, 2011) and patients with spinal cord injury (FVC = 58% of predicted value; *n* = 1) (Xi et al., 2019).

### 3.4 Outcomes of interest

#### 3.4.1 Modalities of RMET

The RMET programmes implemented in the included studies are presented in Table 2. The implementation of RMET was conducted in a variety of settings, including a hospital setting (*n* = 3) (Rigamonti et al., 2014; Salvadego et al., 2017; Villiot‐Danger et al., 2011), an outpatient PR setting (*n* = 1) (Mador et al., 2005) and a patient's home setting (*n* = 6) (Budweiser et al., 2006; Freitag et al., 2018; Koppers et al., 2006; Laurent et al., 2020; Rassler et al., 2007; Scherer et al., 2000). RMET programmes were performed alone (*n* = 6) or in combination with whole‐body exercise training (*n* = 1), usual chest physiotherapy (*n* = 1) or usual care (*n* = 4). The duration of training programmes ranged from 3 weeks to 3 months. The number of sessions ranged from 12 to 20 sessions. The number of RMET sessions per week ranged from 5 to 7 sessions per week. The duration of each session ranged from 10 to 30 min. Some trials reported the intensity of the training (50%–60% MVV and 50%–60% VC) and/or its progression during the training period (from a 5% to 10% increase in MVV or from 1 to 2 breaths/min increase per session). Two studies have proposed a maintenance programme comprising two sessions per week for 4 or 12 months (*n* = 2).

**TABLE 2:** Interventions performed in training and control groups from selected studies.

Author, publication year	Training group (TG)				Control Group (CG)			
	Type	Frequency	Intensity and progression	Duration	Type	Frequency	Intensity and progression	Duration
Koppers et al. (2006)	RMET Home‐based	7 sessions/week 2 sessions/day	60% MVV 60% VC	5 weeks 15 min/session	Incentive flowmeter	7 sessions/week 2 sessions/day	6–7 breaths/min	5 weeks 15 min/session
Mador et al. (2005)	RMET + whole‐body exercise training Outpatient pulmonary rehabilitation		5% to 10% MVV increase	8 weeks 15 min/session	Whole‐body exercise training Cycle ergometer		50% MWR 10% MWR increase	8 weeks
Scherer et al. (2000)	RMET Home‐based	5 sessions/week 2 sessions/day	50% to 60% VC	8 weeks 15 min/session	Incentive volumetric spirometry	5 sessions/week 2 sessions/day	6–8 breaths/min 70% VC	8 weeks 15 min/session
Laurent et al. (2020)	RMET + usual chest physiotherapy Home‐based	5 sessions/week	30% MVV 50% VC 1 breath/min increase/session	3 weeks 30 min/session	Usual chest physiotherapy Airway clearance techniques, deep breathing exercises and thoracic stretching	5 sessions/week		3 weeks 30 min/session
Budweiser et al. (2006)	RMET Home‐based	2 sessions/day	60% MVV 50% VC	3 months 10 min/session	Incentive volumetric spirometry	2 sessions/day	8–10 breaths/min	3 months 10 min/session
Rigamonti et al. (2014)	RMET + usual care (energy‐restricted diet, 90 min/daily aerobic physical activity, psychological counselling and nutritional education) Hospitalization	5 sessions/week	50% to 60% VC	3 weeks	None			
Salvadego et al. (2017)	RMET + usual care Hospitalization	5 sessions/week	50% to 60% MVV	3 weeks	Usual care Caloric restriction, aerobic exercise training, and psychological and nutritional counselling			
Villiot‐Danger et al. (2011)	RMET + usual care Hospitalization	5 sessions/week	50% to 60% MVV 50% VC 1 to 2 breaths/min increase/session	30 min/session	Usual care Low‐caloric diet and physical activity programme, supervised outdoor walking and low‐intensity cycle ergometry without specific control of exercise intensity			
Freitag et al. (2018)	RMET Home‐based	5 sessions/week	50% to 60% MVV 50% to 60% VC	5 weeks 30 min/session	No intervention			
Rassler et al. (2007)	RMET Home‐based	5 sessions/week	50% to 60% MVV 50% to 60% VC	4–6 weeks 30 min/session	None			
Rassler et al. (2011)	RMET	5 sessions/week	50% to 60% MVV 50% to 60% VC	4–6 weeks 30 min/session	None			
Xi et al. (2019)	RMET + usual care	5 sessions/week	50% VC	4 weeks 15–20 min/session	Usual care Passive range of motion, mattress exercise, dynamic sitting balance and upper limb functional training			

Abbreviations: CG, control group; MVV, maximal voluntary ventilation; MWR, maximal work rate; RMET, respiratory muscle endurance training; TG, training group; VC, vital capacity.

#### 3.4.2 Control group

The comparators used for the control in the included studies are presented in Table 2. RMET has been compared with incentive volumetric spirometry (8–12 weeks, 5 sessions per week, 2 daily 10‐min sessions, 70% VC, 6–10 breaths/min; *n* = 2) (Budweiser et al., 2006; Scherer et al., 2000), incentive flowmeter (5 weeks, 7 sessions per week, 2 daily 15‐min sessions, 6–7 breaths/min; *n* = 1) (Koppers et al., 2006), whole‐body exercise training (cycle ergometer, 8 weeks, 50% MWR, 10% MWR increase; *n* = 1) (Mador et al., 2005), chest physiotherapy (3 weeks, 5 sessions per week, 30‐min session, comprising airway‐clearance techniques, deep breathing exercises and thoracic stretching; *n* = 1) (Laurent et al., 2020), usual care (*n* = 3) (Salvadego et al., 2017; Villiot‐Danger et al., 2011; Xi et al., 2019) and no intervention (*n* = 1) (Freitag et al., 2018).

#### 3.4.3 Outcome measures

Respiratory muscle endurance was evaluated using the RET (*n* = 8). Pulmonary function was evaluated through the VC, FVC, FEV_1_, MIP, MEP and MVV (*n* = 4). The assessment of exercise capacity was conducted through the measurement of ${\dot{V}}_{O_{2}\mathrm{peak}}$, MWR, 6MWD and constant‐load exercise endurance time (*n* = 5). The QoL was evaluated through generic or specific questionnaires (*n* = 4). The assessment of symptoms was conducted using dyspnoea and fatigue scales (*n* = 4). Additionally, the incidence of PPCs and the duration of nocturnal NPPV were collected as outcomes (*n* = 2).

#### 3.4.4 Safety and feasibility

Two studies by Rassler et al. (2007, 2011) demonstrated the safety of RMET among patients with myasthenia gravis, including in the patient's home setting. In a study of patients with spinal cord injury, Xi et al. (2019) confirmed the safety of RMET. No adverse events were reported (Rassler et al., 2007, 2011; Xi et al., 2019).

The studies indicated that RMET is a feasible exercise training modality for patients with chronic diseases. Freitag et al. (2018), Laurent et al. (2020) and Rassler et al. (2007, 2011) observed a 100% completion rate, indicating that all patients successfully completed their training programme, including in the patient's home setting. The study by Laurent et al. (2020) among patients eligible for NSCLC resection surgery, was the only one to report an adherence rate for RMET, defined as 75% of the required RMET sessions completed. The reported adherence rate was 86% (Laurent et al., 2020).

#### 3.4.5 Effect of respiratory muscle endurance training

The results from included studies that implemented RMET programmes are presented in Table 3.

**TABLE 3:** Results observed with respiratory muscle endurance training programmes from selected studies.

Author, publication year	Respiratory muscle endurance	Pulmonary function	Exercise capacity	Quality of life	Symptoms	Other outcomes
Koppers et al. (2006)	RET: +299 versus −46 s (*P *< 0.001)		Constant‐load exercise endurance time: +11 versus 0 min (*P *< 0.001) 6MWD: +23 versus −5 m (*P* = 0.020)		Dyspnoea at exercise: −3 versus −1 points (*P* = 0.020)	
Mador et al. (2005)	RET: +18 ± 3 versus +9 ± 3 min (*P* = 0.020)					
Scherer et al. (2000)	RET: +825 ± 170 versus −27 ± 61 s (*P *< 0.001)	MEP: +20 ± 7 versus −6 ± 6 cmH_2_O (*P* = 0.009)	${\dot{V}}_{O_{2}\mathrm{peak}}$: +2.5 ± 0.6 versus −0.3 ± 0.9 mL/min/kg (*P* = 0.015) 6MWD: +58 ± 11 versus +11 ± 11 m (*P* = 0.002)	SF‐12 physical component QoL questionnaire: +9.9 ± 2.7 versus +1.8 ± 2.4 points (*P* = 0.030)		
Laurent et al. (2020)	RET: +229 ± 199 s (TG: *P* = 0.001)					PPCs: 2 versus 10 (*P* = 0.037)
Budweiser et al. (2006)		MIP: +0.86 ± 1.14 versus 0.00 ± 0.80 kPa (*P* = 0.046)	${\dot{V}}_{O_{2}\mathrm{peak}}$: +2.2 ± 3.4 versus −1.7 ± 2.5 mL/min/kg (*P* = 0.014) MWR: +9 ± 15 versus −5 ± 1 W (*P* = 0.043)	SRI physical component QoL questionnaire: +3.3 ± 8.4 versus −6.6 ± 9.0 points (*P* = 0.012)		Nocturnal NPPV time: −0.6 ± 1.2 versus 0.4 ± 0.5 h/day (*P* = 0.010)
Rigamonti et al. (2014)		FVC: +0.5 L (*P *< 0.050)				
Salvadego et al. (2017)			MWR: +18 versus 0 W (TG: *P* = 0.040)		Perceived respiratory exertion at exercise: −2 versus −1 point (TG: *P* = 0.010) Perceived leg exertion at exercise: −1 versus +1 point (TG: *P* = 0.010)	
Villiot‐Danger et al. (2011)	RET: +52 ± 27 versus 0 ± 0% (*P *< 0.001)		6MWD: +54 ± 35 versus +7 ± 31 m (*P *< 0.010)	SF‐36 QoL score: +251 ± 132 versus +84 ± 152 points (*P* = 0.018)	Dyspnoea at exercise: −2 ± 1 versus 1 ± 1 points (*P *< 0.050)	
Freitag et al. (2018)	RET (TG: *P *< 0.001)					
Rassler et al. (2007)	RET: +9 min (*P *< 0.001)					
Rassler et al. (2011)	RET: +9 min (*P *< 0.001)					
Xi et al. (2019)		VC: +10 versus −3% predicted (*P* = 0.016) FEV_1_: +10 versus −4% predicted (*P* = 0.000) MVV: +23 versus −4% predicted (*P* = 0.002)		SGRQ QoL score: −3.0 versus 0.0 points (*P* = 0.000)	Dyspnoea at exercise: −1 versus +2 points (*P* = 0.000)	

Abbreviations: 6MWD, 6‐min walking distance; FEV_1_, forced expiratory volume in the first second; FVC, forced vital capacity; MEP, maximal expiratory pressure; MIP, maximal inspiratory pressure; MVV, maximal voluntary ventilation; MWR, maximal work rate; NPPV, non‐invasive positive‐pressure ventilation; PPCs, pulmonary postoperative complications; QoL, quality of life; RET, respiratory endurance time; SF‐12, 12‐Short Form QoL questionnaire; SF‐36, 36‐Short Form QoL questionnaire; SGRQ, St. George's Respiratory QoL questionnaire; SRI, Severe Respiratory Insufficiency QoL questionnaire; TG, training group; VC, vital capacity; ${\dot{V}}_{O_{2}\mathrm{peak}}$, peak oxygen consumption at exercise.

##### 3.4.5.1 Respiratory muscle endurance

Respiratory muscle endurance training, whether performed as a standalone intervention or in combination with other interventions, increased RET (from +4 to +18 min) (Freitag et al., 2018; Koppers et al., 2006; Laurent et al., 2020; Mador et al., 2005; Rassler et al., 2007, 2011; Scherer et al., 2000; Villiot‐Danger et al., 2011).

##### 3.4.5.2 Pulmonary function

Respiratory muscle endurance training added to usual care increased VC (+10% of the predicted value), FVC (+0.5 L) and FEV_1_ (+10% of the predicted value) (Rigamonti et al., 2014; Xi et al., 2019). Performed alone, it increased MIP (+9 cmH_2_O) or MEP (+20 cmH_2_O) in comparison to incentive volumetric spirometry (Budweiser et al., 2006; Scherer et al., 2000). However, Mador et al. (2005) found that RMET added to whole‐body exercise training had no additional effect on maximal respiratory pressures in comparison to whole‐body exercise training performed alone. RMET added to usual care increased MVV (+23% of the predicted value) in comparison to usual care performed alone (Xi et al., 2019).

##### 3.4.5.3 Exercise capacity

Home‐based RMET performed alone increased ${\dot{V}}_{O_{2}\mathrm{peak}}$ (from +2.2 to +2.5 mL/min/kg) in comparison to incentive volumetric spirometry (Budweiser et al., 2006; Scherer et al., 2000). It increased MWR (from +9 to +18 W) (Budweiser et al., 2006; Salvadego et al., 2017). Performed alone or in combination, it increased 6MWD (from +23 to +58 m) (Koppers et al., 2006; Scherer et al., 2000; Villiot‐Danger et al., 2011). Home‐based RMET increased constant‐load exercise endurance time (+11 min) in comparison to incentive flowmeter (Koppers et al., 2006).

##### 3.4.5.4 Quality of life

Respiratory muscle endurance training, performed alone or in combination, improved the physical component score of the 12‐item Short Form (SF‐12) questionnaire (+10 points), the physical component score of the severe respiratory insufficiency questionnaire (+3 points), the score of the 36‐item Short Form (SF‐36) questionnaire (+251 points), and the score of the St. George's Respiratory (SGRQ) questionnaire (−3 points) (Budweiser et al., 2006; Scherer et al., 2000; Villiot‐Danger et al., 2011; Xi et al., 2019).

##### 3.4.5.5 Symptoms

Respiratory muscle endurance training, performed alone or in combination, reduced dyspnoea at exercise (from −1 to −3 points) (Koppers et al., 2006; Villiot‐Danger et al., 2011; Xi et al., 2019). RMET added to usual care reduced perceived respiratory exertion at exercise (−2 points) and perceived leg exertion at exercise (−1 point) in comparison to usual care performed alone (Salvadego et al., 2017).

##### 3.4.5.6 Other outcomes

Among patients eligible for NSCLC resection surgery, preoperative home‐based RMET added to usual chest physiotherapy decreased the number of PPCs (2 vs. 10) in comparison to usual chest physiotherapy performed alone (Laurent et al., 2020). Among patients with restrictive thoracic disease, it decreased the duration of nocturnal NPPV (−0.6 h/day) in comparison to incentive volumetric spirometry (Budweiser et al., 2006).

### 3.5 Methodological quality of selected studies

The methodological quality of the included studies is presented in Tables 4 and 5. The PEDro scoring system indicated that the overall methodological quality of the included RCTs was rated from low to moderate. It ranged from 3 to 6, with a mean score of 4.5 out of 10. Of the RCTs included, one study was of good quality and the second study was of insufficient quality. The Robins‐1 scoring system indicated that the overall risk of bias of the included non‐RCTs was rated as moderate. All non‐RCT studies were classified with a moderate risk of bias. The main sources of bias in the included studies were insufficient description of the intervention, lack of blinding, missing outcome data, selection of reported results and lack of intention‐to‐treat analysis.

**TABLE 4:** Methodological quality for randomized controlled trials.

Author, publication year	(1) Eligibility	(2) Randomization	(3) Concealed allocation	(4) Similarity at baseline	(5) Blinded patients	(6) Blinded therapists	(7) Blinded assessors	(8) Key outcomes obtained from >85%	(9) Intention to treat	(10) Between‐group statistical comparisons	(11) Both point and variability measures	Total score (/10)
Laurent et al. (2020)	1	1	1	1	0	0	0	0	1	1	1	**6**
Xi et al. (2019)	1	1	0	0	0	0	0	0	0	1	1	**3**

**TABLE 5:** Methodological quality for non‐randomized controlled trials.

Author, publication year	Confounding	Selection	Intervention classification	Deviation from interventions	Missing outcome data	Measurement of outcome	Selection of reported result	Overall risk of bias
Budweiser et al. (2006)	Moderate	Low	Moderate	Low	Low	Low	Low	Moderate
Freitag et al. (2018)	Moderate	Moderate	Moderate	Low	Low	Low	Low	Moderate
Koppers et al. (2006)	Moderate	Low	Moderate	Low	Low	Low	Low	Moderate
Mador et al. (2005)	Moderate	Low	Moderate	Low	Low	Low	Low	Moderate
Rassler et al. (2007)	Moderate	Low	Moderate	Low	Low	Low	Low	Moderate
Rassler et al. (2011)	Moderate	Low	Moderate	Low	Low	Low	Low	Moderate
Rigamonti et al. (2014)	Moderate	Low	Moderate	Low	Low	Low	Low	Moderate
Salvadego et al. (2017)	Moderate	Moderate	Moderate	Low	Low	Low	Low	Moderate
Scherer et al. (2000)	Moderate	Low	Moderate	Low	Low	Low	Low	Moderate
Villiot‐Danger et al. (2011)	Moderate	Low	Moderate	Low	Low	Low	Low	Moderate

## 4 DISCUSSION

This systematic review summarizes the available evidence on RMET among patients with chronic diseases. RMET is an exercise training modality that is both safe and feasible, even in the setting of the patient's home. It increases respiratory endurance time. However, its effect on peak oxygen consumption at exercise, MWR, 6‐min walking distance, QoL, dyspnoea and fatigue remains to be confirmed.

This review was conducted in accordance with the PRISMA guidelines. It included 12 studies, with a total of 257 patients. There was heterogeneity in participants, training modalities and comparators used. The underpowered level of evidence is attributable to the lack of robustness of the original studies, including a lack of description of the intervention, lack of blinding, and missing data. Our review indicates that standardization strategies must be developed before establishing a dose–response relationship or conducting a meta‐analysis for this exercise training modality focused on respiratory muscle endurance.

The main outcome is that RMET increases respiratory muscle endurance, as measured by RET (Freitag et al., 2018; Koppers et al., 2006; Laurent et al., 2020; Mador et al., 2005; Rassler et al., 2007, 2011; Scherer et al., 2000; Villiot‐Danger et al., 2011). Eight of the 12 selected studies reported this parameter as the most consistent outcome. However, to date, no minimal clinically important difference has been established for improvement in RET after RMET. As a result, it is not possible to ascertain the clinical significance of the observed benefit. Further, higher‐quality studies are needed to establish the effect of RMET on RET.

Respiratory muscle endurance training has been shown to increase both MIP and MEP (Budweiser et al., 2006; Scherer et al., 2000). However, Mador et al. (2005) noted that adding RMET to whole‐body exercise training had no additional effect on maximal respiratory pressures. The benefit of RMET on MIP and MEP is still being established against an available minimal clinically important difference, such as among patients with COPD (Beaumont et al., 2023).

The results observed after RMET programmes indicate an increase in exercise capacity (Budweiser et al., 2006; Koppers et al., 2006; Salvadego et al., 2017; Scherer et al., 2000; Villiot‐Danger et al., 2011). However, the effect on ${\dot{V}}_{O_{2}\mathrm{peak}}$ or 6MWD is inconsistent. Nevertheless, these outcomes are predictive factors of adverse outcomes or decreased survival among patients with chronic diseases (Brunelli et al., 2013; Brunelli, Belardinelli et al., 2009; Brunelli, Charloux et al., 2009; Celli et al., 2004). Therefore, it is crucial to evaluate the effect of RMET further on maximal or submaximal exercise capacity.

Studies have noted that RMET improves QoL (Budweiser et al., 2006; Scherer et al., 2000; Villiot‐Danger et al., 2011; Xi et al., 2019) and reduces dyspnoea at exercise (Koppers et al., 2006; Villiot‐Danger et al., 2011; Xi et al., 2019). However, it is challenging to make comparisons between the results on QoL owing to the use of different scales. Nevertheless, these two patient‐reported outcome measures are essential to collect in real life to assess the subjective effect of RMET programmes. Thereby, an effort should be made to include generic and/or specific QoL questionnaires and dyspnoea scales in future studies.

To date, only two studies (Freitag et al., 2018; Rassler et al., 2011) have evaluated the long‐term benefit of RMET. However, both our experience with home‐based RMET and the available literature confirm that maintenance programmes are feasible among patients with chronic diseases. To this end, the implementation of telesupervision in upcoming trials might be a modern means of maintaining the benefit of the initial RMET programmes.

Further investigation is required to gain a deeper understanding of the respiratory muscle adaptations that occur following RMET, as well as in addition to the relationship between respiratory exertion and exercise capacity. Owing to their invasiveness and cost, respiratory muscle biopsies are not commonly used in clinical settings. However, they might prove valuable in understanding the enzymatic and structural mechanisms involved after RMET. Nevertheless, potential explanations are provided in our work. The increase observed in ${\dot{V}}_{O_{2}\mathrm{peak}}$, 6MWD and QoL among patients with moderate to severe COPD might be attributable to a reduction in ventilatory limitation during exercise and might be related to the effect of RMET on RET and dyspnoea (Koppers et al., 2006; Mador et al., 2005; Scherer et al., 2000). RMET has been shown to result in an increase in RET and a decrease in PPCs among patients eligible for NSCLC resection surgery (Laurent et al., 2020). But, no effect of RMET has been noted on ${\dot{V}}_{O_{2}\mathrm{peak}}$, which is the main predictor of postoperative morbimortality in this population (Brunelli et al., 2013; Brunelli, Belardinelli et al., 2009; Brunelli, Charloux et al., 2009). Despite this, RMET might assist in mitigating the physiological stress that occurs in the immediate postoperative period. The improvement observed in MWR, 6MWD and QoL among obese patients can be attributed to the effect of RMET on RET, which coincides with a reduction in fatigue and dyspnoea at exercise (Rigamonti et al., 2014; Salvadego et al., 2017; Villiot‐Danger et al., 2011). Despite the absence of measurement of RET among patients with spinal cord injury, it has been noted that MVV increases after RMET (Xi et al., 2019). Given that MVV is a secondary measure of respiratory muscle endurance, it can be assumed that the improvement in QoL is attributable to an effect of RMET on respiratory endurance and might be mediated by the reduction observed in dyspnoea (Xi et al., 2019). Finally, it is possible that the respiratory metaboreflex might be involved in some of these results, particularly among patients exhibiting ventilatory limitation during exercise. Future explanatory studies should also consider between‐sex and potential ethnic differences.

We acknowledge that our systematic review has several limitations. Firstly, the selected studies were found to be affected by significant biases, including a lack of description and/or missing data. Furthermore, our work included non‐RCTs of a lower methodological quality to provide additional information on the content of the RMET programmes implemented in the original trials. Our analysis revealed that the Clear and Consort grids (Boutron et al., 2008, 2005) for non‐pharmacological trials lack sufficient discriminatory and descriptive power. Instead, we developed our custom data‐extraction grid based on the opinions of a multidisciplinary team of clinicians with expertise in RMET, including physiotherapists, physiologists and thoracic surgeons. This grid allows us to capture better the content of the various RMET programmes implemented and the numerous comparators used.

## 5 CONCLUSIONS

This systematic review shows that RMET increases respiratory endurance among patients with chronic diseases. The populations that benefit the most and the mechanisms involved remain to be investigated. Further high‐quality studies are needed to understand its role, whether it is performed alone or as an add‐on modality to usual pulmonary rehabilitation programmes.

## AUTHOR CONTRIBUTIONS

Hélène Laurent, Marc Filaire and Frédéric Costes contributed to the conception and design of the work. Hélène Laurent, Marc Filaire and Frédéric Costes contributed to the acquisition and analysis. All authors contributed to the drafting, writing, revision and editing of the manuscript. All authors approved the final version of manuscript and agree to be accountable for all aspects of the work in ensuring that questions related to the accuracy or integrity of any part of the work are appropriately investigated and resolved. All persons designated as authors qualify for authorship, and all those who qualify for authorship are listed.

## CONFLICT OF INTEREST

None declared.

## FUNDING INFORMATION

None.
